# A case report on the long‐term use of teduglutide in a pediatric patient with short bowel syndrome

**DOI:** 10.1002/ncp.70023

**Published:** 2025-08-30

**Authors:** Tsuyoshi Sakurai, Hironori Kudo, Nakamura Megumi, Ryo Ando, Takuro Kazama, Ryuji Okubo, Masatoshi Hashimoto, Ko Minoshima, Motoshi Wada

**Affiliations:** ^1^ Department of Pediatric Surgery Tohoku University Hospital Sendai Japan

**Keywords:** intestinal adaptation, intestinal failure, nutrition weaning, parenteral nutrition, pediatric short bowel syndrome, teduglutide

## Abstract

Short bowel syndrome (SBS) is the leading cause of intestinal failure, frequently necessitating long‐term parenteral nutrition (PN). Teduglutide (TED), a glucagon‐like peptide‐2 analog, has demonstrated efficacy in reducing PN dependence in both adults and children. However, long‐term data in pediatric populations remain limited. We present a case of a male child with SBS who underwent extensive small bowel resection at 5 days of age, resulting in a residual small bowel length of 9 cm, with the total colon and ileocecal valve preserved. Despite home PN and multiple interventions, including management of catheter‐related infections and intestinal complications, PN dependence persisted. At the age of 5 years, the patient was enrolled in a TED clinical trial and continued treatment after its commercial approval. During >6 years of TED therapy, gradual and sustained reductions in PN volume and caloric intake were achieved, ultimately resulting in complete weaning from PN. Growth and nutrition status remained stable, and no severe TED‐related adverse events were reported. This is the first known case documenting TED use for >6 years in a pediatric patient with SBS. Although the response was slower than in previously reported cases, long‐term TED administration led to favorable outcomes, including PN independence. This case underscores the potential for TED to support intestinal adaptation over extended periods and highlights the importance of individualized, long‐term treatment strategies in managing severe pediatric SBS.

## BACKGROUND

Intestinal failure (IF) is defined as the inability of the intestine to maintain adequate digestion, nutrient absorption, and fluid balance to support normal growth and health without specialized medical interventions, such as parenteral nutrition (PN).^1^ Short bowel syndrome (SBS) is the leading cause of IF in both adults and children.^1^, ^2^ The quality of life for patients with SBS is significantly impaired because many rely on long‐term total parenteral nutrition (TPN), which carries risks such as intestinal failure–associated liver disease and catheter‐related bloodstream infection (CRBSI).^3^, ^4^, ^5^, ^6^

The primary goal of managing SBS is to promote intestinal adaptation and reduce or eliminate the need for TPN. Recently, teduglutide (TED), a glucagon‐like peptide‐2 analog, was approved for use in adult and pediatric patients with SBS, with its efficacy well documented.^7^, ^8^, ^9^, ^10^ Bioletto et al reported that TED reduced PN requirements by ≥20% in 64% of adult patients after 6 months, 77% after 1 year, and 82% after 2 years. Moreover, PN independence was achieved in 11%, 17%, and 21% of patients at these respective time points.^11^ Post hoc analyses of clinical trials in pediatric populations were published after the approval of TED in the United States, Europe, and Japan.^12^, ^13^, ^14^, ^15^ These studies, spanning 12 to 96 weeks (maximum: 3 years), showed 20% PN volume reductions in 57.1% to 82.1% of patients, consistent with adult outcomes.

Recently, several reports have described TED administration for >4 years, with increasing clarity regarding its effects and adverse events (AEs).^16^, ^17^ However, these reports are limited to adults, and no cases of TED use exceeding 4 years in pediatric patients have been published.

Here, we present a clinical case from our center in Japan demonstrating the long‐term efficacy of TED in a pediatric patient with SBS patient treated for >6 years. This report offers valuable insights into prolonged TED use in pediatric patients. The patient had participated in a TED clinical trial (SHP633‐302/305),^12^ and we have received approval from Takeda Pharmaceutical Company Limited, the trial sponsor, for the use of trial data.

## CASE REPORT

We report a case of a 5‐year‐old male patient with SBS, with no notable family history. He underwent massive intestinal resection because of midgut volvulus with malrotation at 5 days of age. The residual small bowel length was 9 cm, with preservation of the ileocecal valve and the entire colon. He was placed on home parenteral nutrition and referred to our hospital at the age of 2 years. We adjusted his PN volume and caloric intake and initiated treatment with several medications and supplements, including synbiotics, a proton pump inhibitor, and various oral supplements. After transfer, the patient experienced seven episodes of CRBSIs until TED initiation. At age 5, he developed pneumatosis intestinalis, which improved with conservative management. However, recurrence occurred at age 6, and, once stabilized, the patient was admitted for colonoscopy, which led to a diagnosis of eosinophilic gastroenteritis. He was subsequently treated with oral montelukast sodium,^18^ after which no further recurrence was observed. Despite these complications, his growth and development remained on a favorable course, and his PN volume and caloric requirements gradually decreased.

Motivated to improve further, the patient and his family consented to participate in a TED clinical trial (SHP633‐302/305) at age 5, after receiving full information. After the total 1050 days clinical trial, TED became commercially available, and the patient elected to continue treatment. The visit frequency, initially weekly, was gradually extended to once every four weeks during the trial and in postmarketing use it was monthly. Before TED initiation, the duration of TPN use was 5.7 years. Baseline PN volume was 42.4 ml/kg/day, and PN caloric intake was 27.5 kcal/kg/day. A fat emulsion was also administered daily at 0.2 g/kg/day. During the 1003 days before TED initiation, the patient experienced various AEs—including upper respiratory tract inflammation, nonspecific fever, abdominal pain, CRBSIs, and pneumatosis intestinalis, recorded using the same criteria applied post‐TED treatment (Table 1). As part of the screening protocol for TED initiation, a fecal occult blood test was performed instead of colonoscopy because of the patient's young age, in accordance with the clinical trial protocol, and the result was negative. Subsequently, a colonoscopy was performed approximately 6 months after starting TED treatment to evaluate suspected pneumatosis intestinalis, which led to a diagnosis of eosinophilic gastrointestinal disorder. A follow‐up colonoscopy was conducted 1 year after TED initiation to monitor disease progression, and neither examination revealed any polyps.

**Table 1:** Adverse events during the 1003 days prior to and the 2221 days following TED initiation.

Before (1003 days before TED initiation)	*n* (admission)	After (2221 days after TED initiation)	*n* (admission)
CRBSI	7 (7)	CRBSI	3 (3)
URI	6 (2)	URI	3 (2)
Abdominal pain	4 (0)	Abdominal pain	4 (0)
Catheter break	1 (1)	Catheter break	2 (2)
Rash	1 (0)	Rash	1 (0)
Pneumatosis intestinalis	1 (1)	Pneumatosis intestinalis	1 (1)
Headache	1 (0)	Eosinophilic enteritis	1 (1)
Nonspecific fever	2 (2)	Metabolic acidosis	1 (0)

Abbreviations: CRBSI, catheter‐related bloodstream infection; TED, teduglutide; URI, upper respiratory infection.

**Figure 1:**
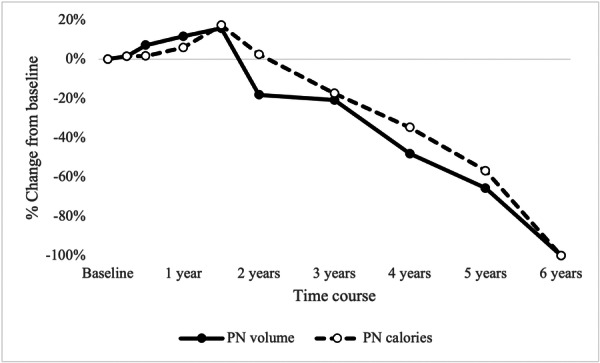
Changes in parenteral nutrition volume and calories over time. Baseline indicates values at the initiation of teduglutide treatment.

**Figure 2:**
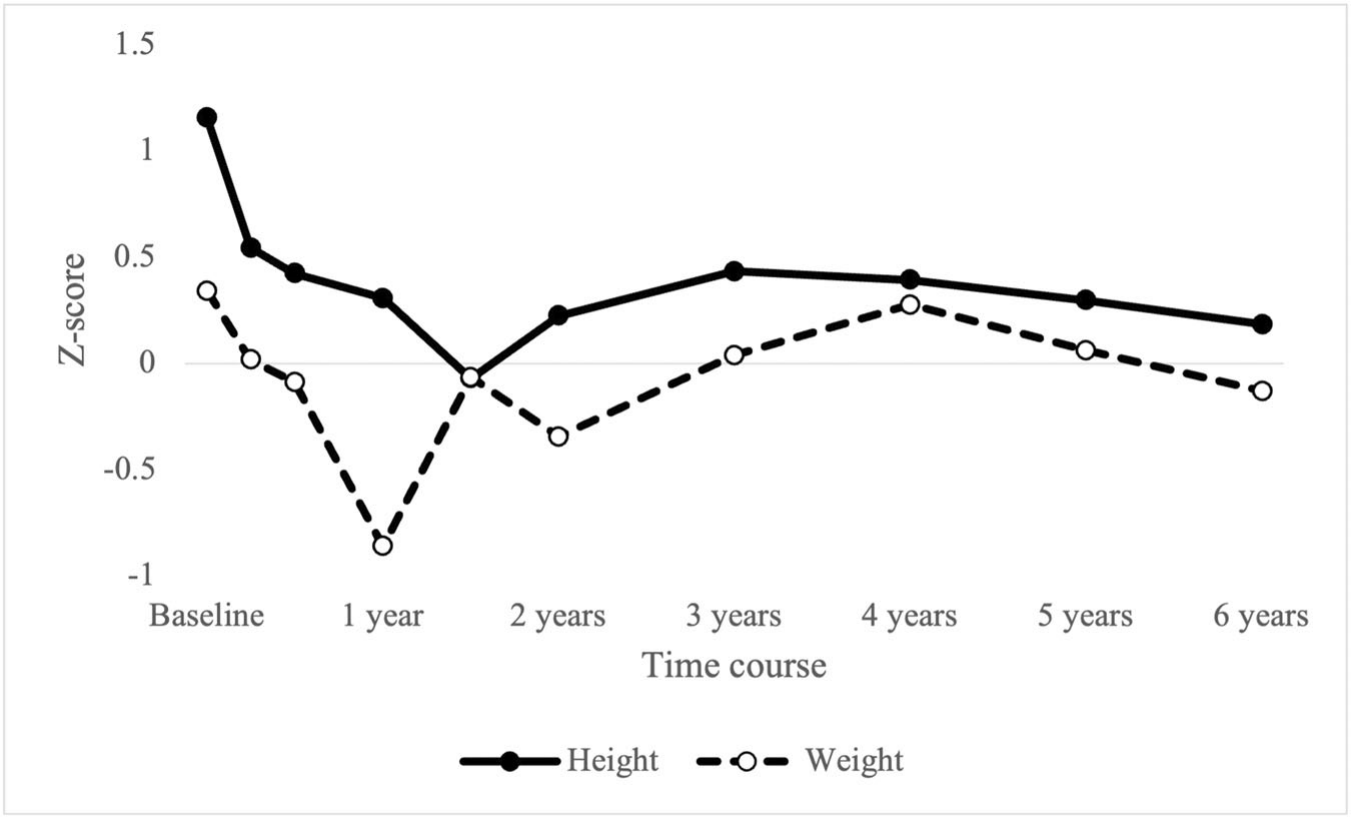
Changes in height and body weight over time. Baseline corresponds to the start of teduglutide therapy.

Figure 1 shows the trends in PN volume and calories following TED initiation. After 2 years, PN volume initially decreased but did not reach the 20% reduction threshold. PN calories remained almost unchanged. By three years post‐treatment, PN volume was reduced by >20%, caloric intake declined, and fat emulsion was reduced by approximately 50%. TED treatment was continued after its commercial approval. The patient remained on TED for >3 additional years and successfully achieved complete weaning from PN after 6 years of treatment. Fat emulsion was discontinued after 4 years, with no significant increase in the triene‐to‐tetraene ratio. Height and weight *z* scores remained relatively stable during TED treatment, indicating appropriate growth for age (Figure 2). During TED administration, no commercially available enteral nutrition formulas or feeding tubes were used; the patient maintained regular oral intake throughout TED treatment. As previously described, oral supplements and medications were continued. Montelukast and loperamide were initiated during the treatment period to manage eosinophilic gastrointestinal symptoms and intestinal motility, respectively. The proton pump inhibitor was discontinued to avoid potential adverse effects of long‐term use, and kanamycin was introduced to support gut microbiota balance. No other significant medication changes were made during this period. Biochemical analyses showed no remarkable changes in liver or renal function, electrolytes, or trace element levels (Table 2). AEs are summarized in Table 1. During the 1003 days preceding TED initiation, there were 23 AEs, 13 of which required hospitalization. In the 2221 days following TED initiation, 17 AEs occurred, with nine requiring hospitalization. The incidence of AEs decreased or remained stable. TED administration was temporarily discontinued because of one CRBSI and one catheter break; treatment was resumed promptly in both instances.

**Table 2:** Summary of blood biochemical data before and during teduglutide (TED) treatment.

Parameter	Before TED initiation, mean (range)	During TED treatment, mean (range)
AST, U/L	39.5 (29–93)	33.6 (21–73)
ALT, U/L	32.0 (16–117)	39.7 (21–131)
TP, g/dl	6.4 (6.1–7.0)	6.6 (4.9–7.2)
Serum albumin level, g/dl	4.2 (3.6–4.5)	4.2 (2.8–5.0)
TG	48.5 (27–69)	74.8 (36–161)
Tchol	78.0 (65–90)	82.3 (46–116)
Zinc, mcg/dl	66.5 (57–71)	72.3 (60–84)
Copper, mcg/dl	104.8 (91–122)	110.2 (86–143)
Selenium, mcg/L	80.7 (64–92)	93.7 (45–119)
Citrulline, μmol/L	11.25 (9.1–13.4)	10.9 (8.3–14.1)
TT ratio	0.015 (0.01–0.02)	0.03 (0.01–0.05)

*Note*: “Before TED” refers to laboratory values collected during the 1003 days prior to TED initiation. “During TED” refers to values obtained over the 2221 days of ongoing TED treatment, including after parenteral nutrition discontinuation.

Abbreviations: ALP, alkaline phosphatase; ALT, alanine transaminase; AST, aspartate transaminase; Tchol, total cholesterol; TED, teduglutide; TG, triglyceride; TP, total protein; TT, ratio triene/tetraene ratio

This case report was approved by the Ethics Committee of Tohoku University Hospital (Approval No. 29924) and performed in accordance with the ethical standards of the Declaration of Helsinki 1964 and its later amendments.

## DISCUSSION

We present a case evaluating the efficacy of TED in a pediatric patient with SBS. Although this is a single case report, there are few published studies focused on long‐term TED use in pediatric populations. To our knowledge, this is the first reported case worldwide of TED administration exceeding 6 years in a pediatric patient. Reports on the long‐term effects of TED beyond 2 years in children are scarce, with only five studies identified in our review^19^, ^20^, ^21^, ^22^, ^23^ (Table 3). The median treatment duration in these studies was approximately 2 years, and all reported positive outcomes, with ≥20% PN reduction in 62.0% to 82.1% of cases and PN weaning rates ranging from 15.4% to 35.7%. Although our patient responded more slowly than those in previous reports, PN independence was eventually achieved without any serious long‐term AEs.

**Table 3:** Publications with >2 years of TED treatment.

Publication	Study design	*n*	Median age (year)	Treatment duration	Result (20% reduction/weaned off)	AE (any related to TED/severe)
Nucci et al 2025^18^	Retrospective multicenter study	14	9.5 (IQR: 5.8–11)	840 days (IQR: 425–1530 days)	nm/5	nm
Wales et al 2024^19^	Post hoc analysis	78	4 (range: 0.5–15.0)	96 weeks	82.1%/21.8%	24.4%/6.4%
Germán‐Díaz et al 2024^20^	Prospective multicenter study	31	2.3 (IQR: 1.4–4.9)	19months (IQR: 12–36 months)	24/9	6/0
Guz‐Mark et al 2022^21^	Retrospective multicenter study	13	6 (IQR: 4.7–7)	18 months (IQR: 12–30 months)	8/2	nm/0
Falco et al 2022^22^	Case report	1	11	2.5 years	1/1	0/0

Abbreviations: AE, adverse event; IQR, interquartile range; nm, not mentioned; TED, teduglutide.

Gigola et al published the only systematic review to date evaluating TED use in patients <18 years of age, released in 2022.^24^ They reported that, after a median of 24 weeks of TED treatment, 67% of patients achieved a reduction in PN requirements, and 16% were fully weaned from PN. These authors posited that the variability in response time to TED is likely attributable to heterogeneity in underlying diseases and patient characteristics.^12^, ^21^, ^22^, ^25^ In our case, the patient had a residual small bowel length of <9 cm and required prolonged high‐volume PN, both factors indicating severe SBS. Additionally, previous studies have identified the presence of the colon and ileocecal valve as negative predictors of early response in adults.^11^, ^26^, ^27^ Racial and systemic healthcare differences may also influence treatment outcomes.^20^, ^28^ In Japan, patients with SBS have access to extensive medical support with minimal out‐of‐pocket costs, allowing for optimal care, including home PN.^29^ Despite the lack of a marked increase in citrulline levels, previously associated with TED response,^21^, ^30^ our patient still achieved favorable outcomes, suggesting that mechanisms other than mucosal mass expansion, such as improved intestinal barrier function, motility modulation, or enhanced nutrient absorption, may be involved.^31^, ^32^, ^33^ AEs observed in this case were consistent with those in previous reports, and there were no major differences in their incidence before and after TED initiation.^12^, ^34^, ^35^, ^36^

Our findings suggest that longer treatment durations may be necessary for an optimal response, a trend also observed in adults.^8^, ^9^, ^13^ Boluda et al noted the difficulty in determining the optimal duration of TED therapy in pediatric SBS.^37^ Wales et al recommended maintaining treatment for at least 1 year, whereas Diamanti et al proposed discontinuing TED in patients who do not achieve a ≥20% PN reduction within 6 months.^20^, ^36^ Although prolonged administration is often limited by high costs,^38^, ^39^ our findings suggest that treatment for >3 years may be required, especially in cases with a delayed but clear response.

The necessity of continuing TED after the discontinuation of PN remains controversial. According to a survey by Harpain et al, only 27% of healthcare providers anticipated the need for PN reinitiation following the cessation of TED, suggesting that most do not expect redependence.^40^ However, Compher et al reported that 40.5% of patients required increased PN volumes within 12 months after discontinuing TED, whereas Zaczek et al observed PN volume increases in 92.3% of patients over a 9‐year follow‐up period.^41^, ^42^ Both authors emphasized the importance of stratifying patients based on their long‐term dependency on TED. In this context, continued TED treatment after PN discontinuation may be justified, particularly in unique cases such as ours, in which a delayed yet clear clinical response was observed.

This case report has certain limitations. First, we lacked a standardized protocol for PN reduction, which may have contributed to variability in response time and treatment outcomes compared with other reports. Nevertheless, PN reduction appeared appropriate because the patient maintained stable growth parameters throughout treatment. Second, part of this case occurred during a clinical trial, and, third, the absence of a control group makes it challenging to definitively attribute improvements to TED, particularly since gradual PN reductions began before TED initiation. However, given the patient's prolonged dependence receiving PN for >5 years, spontaneous adaptation was unlikely without pharmacologic TED intervention.

Our case suggests that TED can be effective in pediatric SBS even in severe, long‐standing cases, although a therapeutic response may require extended treatment duration. According to a multicenter pediatric study,^20^ earlier initiation of TED is associated with improved outcomes. This suggests that timely intervention during early childhood may enhance intestinal adaptation in pediatric patients with intestinal failure. Because there are currently no long‐term real‐world data on TED use in pediatric SBS, further prospective clinical studies are warranted to better define treatment duration, predictors of response, and safety over time.

## AUTHOR CONTRIBUTIONS

Tsuyoshi Sakurai contributed to the conception and design of the research; Tsuyoshi Sakurai, Hironori Kudo, Nakamura Megumi, and Ryo Ando contributed to the acquisition of the data; Tsuyoshi Sakurai drafted the manuscript; and all authors revised the manuscript, agreed to be fully accountable for ensuring the integrity and accuracy of the work, and read and approved the final manuscript.

## CONFLICT OF INTEREST STATEMENT

Motoshi Wada has received lecture and advisory honoraria from Takeda Pharmaceutical Company Limited, (the manufacturer of teduglutide). The remaining authors declare no conflicts of interest.

## INFORMED CONSENT STATEMENT

Written informed consent was obtained from the patient's legal guardian for publication of this case report.

## References

[1] Pironi L , Arends J , Baxter J , et al. ESPEN endorsed recommendations. Definition and classification of intestinal failure in adults. Clin Nutr. 2015;34(2):171‐180. 25311444 10.1016/j.clnu.2014.08.017

[2] Pironi L , Arends J , Bozzetti F , et al. ESPEN guidelines on chronic intestinal failure in adults. Clin Nutr. 2016;35(2):247‐307. 26944585 10.1016/j.clnu.2016.01.020

[3] Caporilli C , Giannì G , Grassi F , Esposito S . An overview of short‐bowel syndrome in pediatric patients: focus on clinical management and prevention of complications. Nutrients. 2023;15(10):2341. 37242224 10.3390/nu15102341 PMC10221592

[4] Winkler M , Tappenden K . Epidemiology, survival, costs, and quality of life in adults with short bowel syndrome. Nutr Clin Pract. 2023;38(suppl 1):S17‐S26. 37115027 10.1002/ncp.10964

[5] Winkler MF , Smith CE . Clinical, social, and economic impacts of home parenteral nutrition dependence in short bowel syndrome. JPEN J Parenter Enteral Nutr. 2014;38(1 suppl):32S‐37S. 24418898 10.1177/0148607113517717

[6] Kudo H , Wada M . Pediatric intestinal rehabilitation. Curr Opin Organ Transplant. 2023;28(3):237‐241. 37053076 10.1097/MOT.0000000000001062

[7] Jeppesen PB , Pertkiewicz M , Messing B , et al. Teduglutide reduces need for parenteral support among patients with short bowel syndrome with intestinal failure. Gastroenterology. 2012;143(6):1473‐1481.e3. 22982184 10.1053/j.gastro.2012.09.007

[8] Seidner DL , Fujioka K , Boullata JI , Iyer K , Lee HM , Ziegler TR . Reduction of parenteral nutrition and hydration support and safety with long‐term teduglutide treatment in patients with short bowel syndrome‐associated intestinal failure: STEPS‐3 Study. Nutr Clin Pract. 2018;33(4):520‐527. 29761915 10.1002/ncp.10092

[9] Nakamura S , Wada M , Mizushima T , et al. Efficacy, safety, and pharmacokinetics of teduglutide in adult Japanese patients with short bowel syndrome and intestinal failure: two phase III studies with an extension. Surg Today. 2023;53(3):347‐359. 36201060 10.1007/s00595-022-02587-4 PMC9950205

[10] Joly F , Seguy D , Nuzzo A , et al. Six‐month outcomes of teduglutide treatment in adult patients with short bowel syndrome with chronic intestinal failure: a real‐world French observational cohort study. Clin Nutr. 2020;39(9):2856‐2862. 31932048 10.1016/j.clnu.2019.12.019

[11] Bioletto F , D'Eusebio C , Merlo FD , et al. Efficacy of teduglutide for parenteral support reduction in patients with short bowel syndrome: a systematic review and meta‐analysis. Nutrients. 2022;14(4):796. 35215445 10.3390/nu14040796 PMC8880479

[12] Chiba M , Masumoto K , Kaji T , et al. Efficacy and safety of teduglutide in infants and children with short bowel syndrome dependent on parenteral support. J Pediatr Gastroenterol Nutr. 2023;77(3):339‐346. 37364133 10.1097/MPG.0000000000003867 PMC10412081

[13] Fifi A , Raphael BP , Terreri B , Uddin S , Kaufman SS . Effects of teduglutide on diarrhea in pediatric patients with short bowel syndrome‐associated intestinal failure. J Pediatr Gastroenterol Nutr. 2023;77(5):666‐671. 37889619 10.1097/MPG.0000000000003922 PMC10583903

[14] Chen K , Mu F , Xie J , et al. Impact of teduglutide on quality of life among patients with short bowel syndrome and intestinal failure. JPEN J Parenter Enteral Nutr. 2020;44(1):119‐128. 31006876 10.1002/jpen.1588 PMC7004164

[15] Carter BA , Cohran VC , Cole CR , et al. Outcomes from a 12‐week, open‐label, multicenter clinical trial of teduglutide in pediatric short bowel syndrome. J Pediatr. 2017;181:102‐111.e5. 27855998 10.1016/j.jpeds.2016.10.027

[16] Joly F , Jezerski D , Pape UF , et al. Real‐world experience of teduglutide use in adults with short bowel syndrome: a seven‐year international multicenter survey. Clin Nutr. 2025;47:54‐67. 39986179 10.1016/j.clnu.2025.01.026

[17] Mazzuoli S , Regano N , Lamacchia S , Silvestri A , Guglielmi FW . Forty‐eight months outcomes of teduglutide treatment in adult stable patients with short bowel syndrome and home parenteral nutrition dependence: a real‐world Italian single‐center observational cohort study. Nutrition. 2025;131:112640. 39689615 10.1016/j.nut.2024.112640

[18] Tien FM , Wu JF , Jeng YM , et al. Clinical features and treatment responses of children with eosinophilic gastroenteritis. Pediatr Neonatol. 2011;52(5):272‐278. 22036223 10.1016/j.pedneo.2011.06.006

[19] Nucci AM , Bashaw H , Kirpich A , Rudolph J . Retrospective review of growth in pediatric intestinal failure after weaning from parenteral nutrition. Nutr Clin Pract. 2025;40(1):176‐187. 39263924 10.1002/ncp.11209 PMC11713205

[20] Wales PW , Hill S , Robinson I , et al. Long‐term teduglutide associated with improved response in pediatric short bowel syndrome‐associated intestinal failure. J Pediatr Gastroenterol Nutr. 2024;79(2):290‐300. 38873891 10.1002/jpn3.12276

[21] Germán‐Díaz M , Alcolea A , Cabello V , et al. Early use of teduglutide in paediatric patients with intestinal failure is associated with a greater response rate: a multicenter study. Eur J Pediatr. 2024;183(8):3173‐3182. 38664251 10.1007/s00431-024-05577-5

[22] Guz‐Mark A , Hino B , Berkowitz D , et al. The variable response to teduglutide in pediatric short bowel syndrome: a single country real‐life experience. J Pediatr Gastroenterol Nutr. 2022;75(3):293‐298. 35730756 10.1097/MPG.0000000000003541

[23] Falco EC , Lezo A , Calvo P , et al. Case report: morphologic and functional characteristics of intestinal mucosa in a child with short bowel syndrome after treatment with teduglutide: Evidence in favor of GLP‐2 analog safety. Front Nutr. 2022;9:866048. 35811959 10.3389/fnut.2022.866048 PMC9261410

[24] Gigola F , Cianci MC , Cirocchi R , et al. Use of teduglutide in children with intestinal failure: a systematic review. Front Nutr. 2022;9:866518. 35774551 10.3389/fnut.2022.866518 PMC9237607

[25] Micic D , Robinson I , Kidd T , Terreri B , Raphael BP . Teduglutide improves liver chemistries in short bowel syndrome‐associated intestinal failure: Post hoc analysis. Nutr Clin Pract. 2024;39(3):634‐640. 38491966 10.1002/ncp.11139

[26] Pironi L . Translation of evidence into practice with teduglutide in the management of adults with intestinal failure due to short‐bowel syndrome: a review of recent literature. JPEN J Parenter Enteral Nutr. 2020;44(6):968‐978. 31802516 10.1002/jpen.1757

[27] Chen K , Joly F , Mu F , et al. Predictors and timing of response to teduglutide in patients with short bowel syndrome dependent on parenteral support. Clin Nutr ESPEN. 2021;43:420‐427. 34024550 10.1016/j.clnesp.2021.03.011

[28] Lambe C , Talbotec C , Kapel N , et al. Long‐term treatment with teduglutide: a 48‐week open‐label single‐center clinical trial in children with short bowel syndrome. Am J Clin Nutr. 2023;117(6):1152‐1163. 37270289 10.1016/j.ajcnut.2023.02.019

[29] Sawakami T . Current status of specific pediatric chronic diseases in Japan: national measures, disease types, treatment availability, copayment assistance, and research. Intractable Rare Dis Res. 2021;10(4):283‐287. 34877241 10.5582/irdr.2021.01145 PMC8630460

[30] Kocoshis SA , Merritt RJ , Hill S , et al. Safety and efficacy of teduglutide in pediatric patients with intestinal failure due to short bowel syndrome: a 24‐week, phase III study. JPEN J Parenter Enteral Nutr. 2020;44(4):621‐631. 31495952 10.1002/jpen.1690 PMC7318247

[31] Martin GR , Beck PL , Sigalet DL . Gut hormones, and short bowel syndrome: the enigmatic role of glucagon‐like peptide‐2 in the regulation of intestinal adaptation. World J Gastroenterol. 2006;12(26):4117‐4129. 16830359 10.3748/wjg.v12.i26.4117 PMC4087358

[32] Meier JJ , Nauck MA , Pott A , et al. Glucagon‐like peptide 2 stimulates glucagon secretion, enhances lipid absorption, and inhibits gastric acid secretion in humans. Gastroenterology. 2006;130(1):44‐54. 16401467 10.1053/j.gastro.2005.10.004

[33] Hvistendahl MK , Naimi RM , Enevoldsen LH , Madsen JL , Fuglsang S , Jeppesen PB . Effect of Glepaglutide, a long‐acting glucagon‐like peptide‐2 analog, on gastrointestinal transit time and motility in patients with short bowel syndrome: findings from a randomized trial. JPEN J Parenter Enteral Nutr. 2020;44(8):1535‐1544. 32022286 10.1002/jpen.1767

[34] Oliveira SB , Kocoshis SA . Teduglutide for the treatment of short bowel syndrome: a double‐edged sword? Am J Clin Nutr. 2023;117(6):1057‐1058. 37270286 10.1016/j.ajcnut.2023.04.009

[35] Puello F , Wall E , Herlitz J , Lozano ES , Semrad C , Micic D . Long‐term outcomes with teduglutide from a single center. JPEN J Parenter Enteral Nutr. 2021;45(2):318‐322. 32391948 10.1002/jpen.1838

[36] Diamanti A , Lezo A , D'Antiga L , et al. Teduglutide in pediatric intestinal failure: a position statement of the Italian society of pediatric gastroenterology, hepatology and nutrition (SIGENP). Dig Liver Dis. 2022;54(10):1320‐1327. 35654733 10.1016/j.dld.2022.04.028

[37] Ramos Boluda E , Redecillas Ferreiro S , Manrique Moral O , et al. Experience with teduglutide in pediatric short bowel syndrome: first real‐life data. J Pediatr Gastroenterol Nutr. 2020;71(6):734‐739. 32804906 10.1097/MPG.0000000000002899

[38] Raghu VK , Rudolph JA , Smith KJ . Cost‐effectiveness of teduglutide in pediatric patients with short bowel syndrome: Markov modeling using traditional cost‐effectiveness criteria. Am J Clin Nutr. 2021;113(1):172‐178. 33021637 10.1093/ajcn/nqaa278 PMC9630124

[39] Cucinotta U , Acunzo M , Payen E , et al. The impact of teduglutide on real‐life health care costs in children with short bowel syndrome. J Pediatr. 2024;272:113882. 38135030 10.1016/j.jpeds.2023.113882

[40] Harpain F , Milicevic S , Howard L , Biedermann P , Pape UF . Management patterns of teduglutide use in short bowel syndrome: a survey of 70 healthcare professionals. Nutrients. 2024;16(21):3762. 39519595 10.3390/nu16213762 PMC11547446

[41] Compher C , Gilroy R , Pertkiewicz M , et al. Maintenance of parenteral nutrition volume reduction, without weight loss, after stopping teduglutide in a subset of patients with short bowel syndrome. JPEN J Parenter Enteral Nutr. 2011;35(5):603‐609. 21825090 10.1177/0148607111414431

[42] Zaczek Z , Jurczak‐Kobus P , Panczyk M , et al. Changes in parenteral nutrition requirements and BMI in patients with parenteral nutrition‐dependent short bowel syndrome after stopping teduglutide‐9 years of follow‐up. Nutrients. 2022;14(8):1634. 35458196 10.3390/nu14081634 PMC9024979

